# Development of a Small Footprint Device for Measuring Electrodermal Activity in the Palm of the Hand

**DOI:** 10.2478/joeb-2022-0021

**Published:** 2023-01-14

**Authors:** Åsmund Aukrust, Leah Marie Foseid, Kristiane Holm

**Affiliations:** 1 Department of Clinical and Biomedical Engineering, Oslo University Hospital, Oslo, Norway; 2 Department of Physics, University of Oslo, Oslo, Norway

**Keywords:** Bioimpedance, electrodermal activity, electrode placement, signal processing

## Abstract

This paper describes the proof of concept for a wearable device that measures skin conductance, to provide a way of quantifying an individual’s physiological stress response to external stimuli. Important goals of the project were to have reliable measurements that correlate with the external stimuli, as well as a small footprint and low power consumption to facilitate battery powered operation.

These goals were accomplished using a STM32L476 micro-controller to generate an AC sine voltage across two solid gel electrodes placed in the palm of the hand, converting the resulting current to a voltage with a trans-impedance amplifier, which was then sampled and processed digitally in a lock-in amplifier, to eliminate signals differing from the desired (reference) frequency and phase. The output of the lock-in amplifier represents the skin conductance and was transmitted over USB to a computer with software for serial capture.

## Introduction

While somatic medicine has virtually been revolutionized by technological developments the last decades, very little has happened in this area within the mental health sector. Hence, there is a need for sensor systems that can monitor physiological parameters linked to the emotional or mental state of a person. Electrodermal activity (EDA) has been shown to be a useful proxy for activity in the sympathetic nervous system and is strongly linked to the level of arousal or psychological stress (Trondstad et al., 2022).

This paper reports on the contstruction of a small footprint device for measuring EDA. The project was carried out as a collaboration between bachelor students at Oslo Metropolitan University and the company Nordic Neurotech AS, Norway. The measurements were carried out by using two solid gel electrodes placed in the palm of the hand, and an applied controlled AC sine signal. Employing a trans-impedance amplifier the resulting current was converted to a voltage, which was sampled. The signal was then multiplied with the applied signal. This product was integrated over a given number of periods to represent the electrical conductance of the skin and then displayed graphically on a computer.

In addition to describing the construction of the instrument, this article deals with whether it is possible to measure EDA on the wrist and e.g., implement the EDA device into a smartwatch attached to the wrist, or if EDA is only possible to measure by having the electrodes placed in the palm of the hand.

## Materials and methods

**Figure 1:**
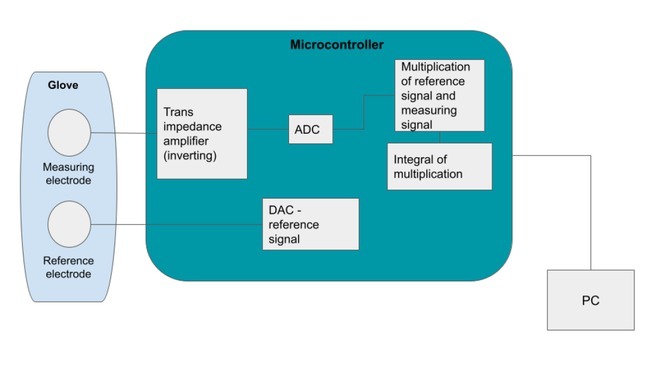
Functional design specification

The device was based on an STM32L476 microcontroller on a minimal breakout board with USB connection, connected to two solid gel electrodes in the palm of the hand, as detailed in figure 1.

## Measuring frequency

An AC signal was used to avoid electrode polarization and to reduce the applied voltage, as recommended in the literature (Recommendations, 2012). To make sure the device is measuring skin conductance, the measuring frequency was set to 22 Hz (Martinsen et al., 2015).

## Instrument design

Conductance is measured with the help of an AC signal applied to two solid gel electrodes placed in the palm of the hand. The resulting current is converted to a voltage using a trans-impedance amplifier and processed through a lock-in amplifier to produce the skin conductance.

In the investigation phase, we concluded with these minimum requirements for the circuit:

- Low power consumption
- SMD components to minimize size
- Bluetooth or other wireless data transmission
- Low price

As price and power consumption were important factors, a solution using as few as possible separate components was decided on, where multiplication and integration are done digitally in a microcontroller. This bypasses the need for expensive analog circuitry and allows for future expansion of functionality to include wireless transmission.

## Microcontroller

Requirements for selecting a microcontroller were:

- Minimum 12-bit ADC to get sufficient resolution
- DAC to generate the AC sine signal
- Low power consumption
- Processing power
- Price

A microcontroller in the STM32L4 family fits these requirements well. This series consists of 32-bit microcontrollers optimized for low power consumption, with 12-bit ADC, 80 MHz ARM Cortex-M4 CPU with floating point unit and digital signal processor (STMicroelectronics). A development/breakout board based on STM32L476RET6 was chosen as it was readily available and comes with a wide variety of peripherals.

**Figure 2:**
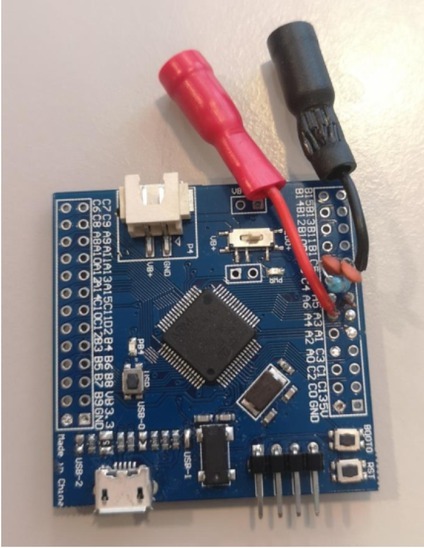
STM32L476 breakout board with feedback components and electrode connections

The development platform STM32CubeIDE was used to configure peripherals and generate code for further development. The code was edited to provide the desired functionality, then compiled and uploaded to the microcontroller.

**Figure 3:**
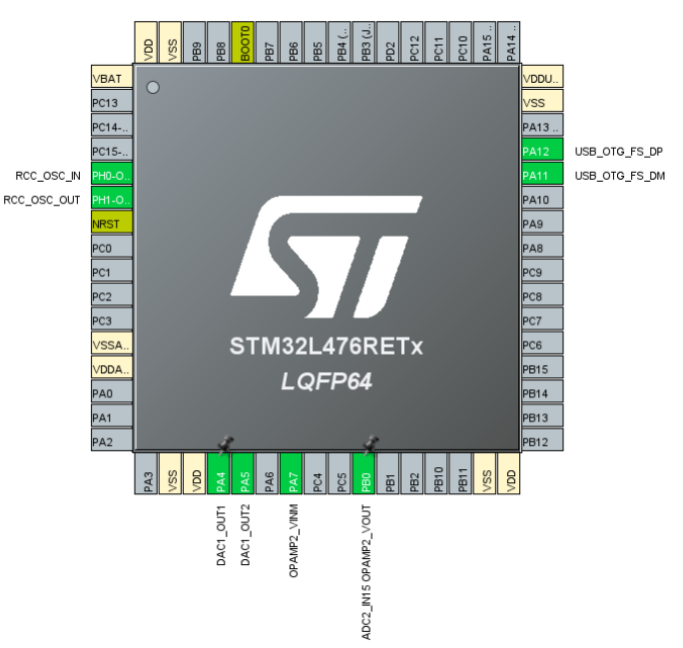
Pin configuration in STM32CubeIDE

The functionality included in the code:

- Generate a sine wave with a frequency of 22 Hz an amplitude of 50-150 mV rms using the DAC
- Integrated operational amplifier configured as a trans-impedance amplifier to convert the current from the electrodes into a voltage
- Sampling the output of the trans-impedance amplifier with the ADC, using a sampling frequency of 5632 samples per second
• Multiplication of each sampled value with the corresponding value from the DAC
- Integration of the multiplied signal over five periods
- Transmission of the obtained value over USB

To generate a sine wave with the desired frequency, a timer with a frequency of 22 *×* 256 = 5632 Hz was configured to create a sine wave consisting of 256 points in each period. DAC 1 channel 1 of the microcontroller was configured as the sine wave using circular DMA, while DAC 1 channel 2 was configured as reference for the trans-impedance amplifier and connected to the non-inverting input of one of the integrated opamps. Circular DMA is useful, as in this way the microcontroller only needs to store DAC values for one period of the sine wave. The ADC was configured to sample the output of this integrated opamp with a sampling frequency of 5632 Hz using the same timer. ADC interrupts were enabled, thus the code can be executed from the callback function each time a sampling is finished. The inverting input of the trans-impedance opamp was connected to the electrodes. USB was enabled and configured for serial communication. External passive components were placed over the opamp, providing a feedback resistance of 680 kΩ and a capacitance of 6.9 nF.

## Electrodes, data transmission, capture and visualization

The most suitable placement of the electrodes for EDA measurements is in the palm of the hand (Grimnes and Martinsen, 2015). The density of sweat glands is highest in the palm and the glands are innervated by the sympathetic nervous system. Furthermore, this placement was justified by completing various tests with different electrode placements.

Sweating in the wrist, however, is not psychologically conditioned, and the density of sweat glands is comparatively low. As a result, the measurements here will presumably be less accurate and will not illustrate the activation of the sympathetic nervous system.

H124SG Covidien EMG solid gel electrodes were used, as these provided a suitable contact area with a diameter of 23.9 mm. CoolTerm 1.9.1.964 was used to receive and store serial data, while Kst 2 was used to plot data in real time.

## Design of the glove

In a future application, we speculate that the electrodes can be made of conducting fabric and implemented inside a glove. A prototype glove was hence designed as a pulse glove without fingers. This was done to assure that the glove could be worn by anyone, regardless of hand size. The wires that goes from the electrodes in the palmar skin sites and to the instrument is sewn into the glove, to minimize noise by movement. However, the "button head on the wires" was not completely secured, to allow for certain adjustments when applying the electrodes to the palm.

## Reference test

As a reference test for comparison with regards to electrode placement, electrodes were placed around the wrist as pictured below

**Figure 4:**
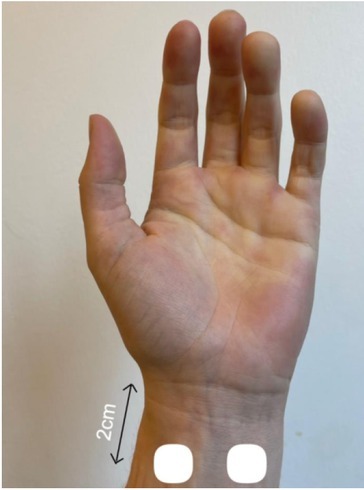
Electrode placement for the reference test

## Informed consent

Informed consent has been obtained from all individuals included in this study.

## Ethical approval

The research related to human use has been complied with all relevant national regulations, institutional policies and in accordance with the tenets of the Helsinki Declaration, and has been approved by the authors’ institutional review board or equivalent committee.

## Results

**Figure 5:**
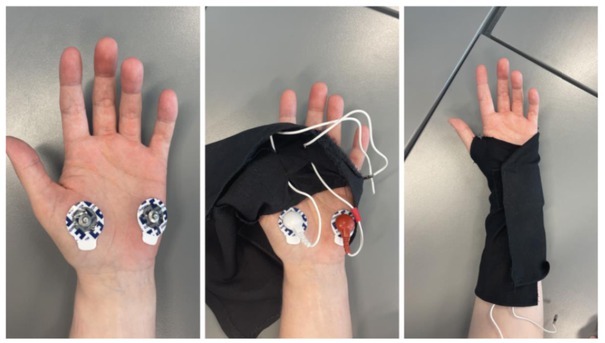
Photo of electrode placement and glove

Five people were tested under the same conditions, undergoing the same exposure from VR goggles. The exposure consisted of a five minute guided meditation video followed by a virtual base jump from a mountain cliff and then a repetition of the first meditation video. The results from the tests show graphically how the device measures change in conductance differently with regard to the placement of the electrodes.

**Figure 6:**
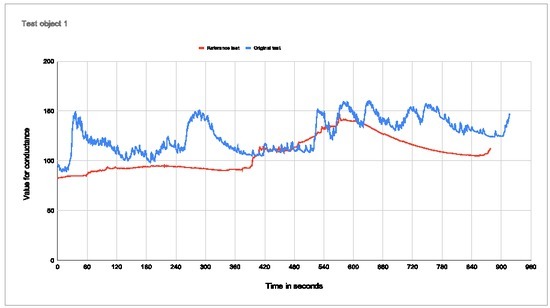
Plot of conductance for subject 1 Percentage change with electrodes in palm: 44,37% Percentage change with electrodes on arm: 43,43%

Test subject 1 stated afterward that one of the electrodes had come loose during the test with electrodes in the palm due to sweat in the hand. The results show that the conductance increases during the base jump, both with electrodes in the palm and on the arm, but that there is some latency and a much lower percentage increase than we will see in the next test subjects. The measurement is somewhat noisy.

**Figure 7:**
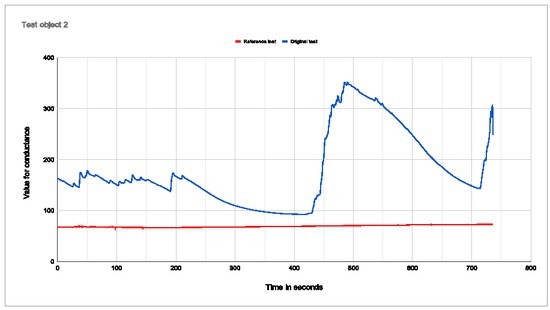
Plot of conductance for test subject 2 Percentage change with electrodes in palm: 73,78% Percentage change with electrodes on arm: 13,48%

Test subject 2 stayed calm and focused throughout the test, and replied that he/she became more relaxed during the test and the meditation. The person also sat completely still. The curve with electrode placement in the palm is clear and shows an abrupt increase in conductance when exposed to the virtual base jump. The conductance decreases again during the last meditation. With the electrodes placed on the arm, there is barely any change in conductance.

**Figure 8:**
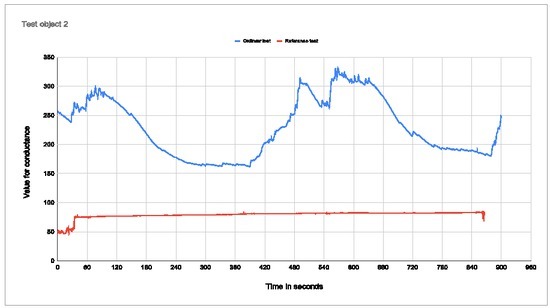
Plot of conductance for test subject 3 Percentage change with electrodes in palm: 51,16% Percentage change with electrodes on arm: 44,61%

Test subject 3 became frustrated with the voice that guided the meditation, and thus lost focus during the test. It took time for the person to calm down before the base jump, and one can see that there is latency from the start of the base jump video until you get a clear result. During the last meditation part, the conductance curve goes gradually back towards the starting baseline.

**Figure 9:**
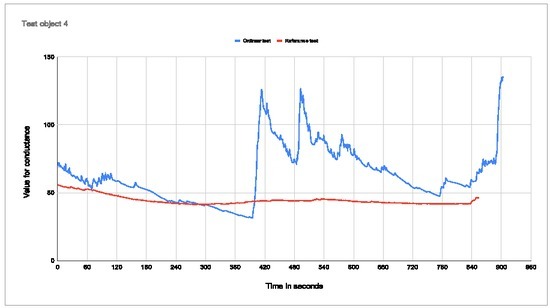
Plot of conductance for test subject 4 Percentage change with electrodes in palm: 76,81% Percentage change with electrodes on arm: 26,13%

Test subject 4 reports that he/she easily becomes unfocused and noticed other things in the VR world, e.g. poor graphics and "glitches". There is a distinct increase in conductance as the base jump video kicks in when electrodes are placed in the palm. With electrodes on the wrist, there is little increase in conductance. The second time the conductance increases is when the base jumper is heading towards a rock wall in the middle of the video. The graph shows that the conductance of the person was already on the way down in the last part of the base jump.

Test subject 5 needed adjustments at the start and during the test. The impact was apparently not as large as for the previous test subjects, which you can see as the percentage change is low with the electrodes placed in the palm. After the jump, the person quickly drops to the same conductance value as before the jump.

**Figure 10:**
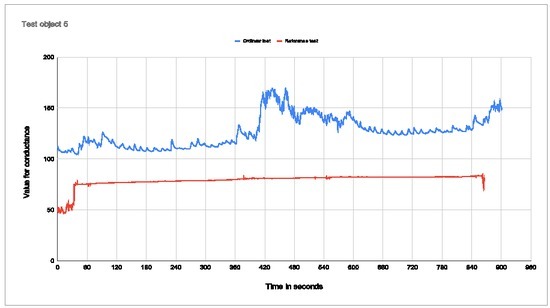
Plot of conductance for test subject 5 Percentage change with electrodes in palm: 38,18% Percentage change with electrodes on arm: 44,61%

## Discussion

In this article, the main focus has been to construct an EDA device and to investigate the possibility to measure skin conductance at the wrist. The purpose of this is to see if it is possible to implement conductance measurements in, e.g., a smartwatch.

As presented in the test results, with the electrodes placed in the palm and on the wrist, the placement of electrodes is important for the ability to measure a change in conductance. For all the measurements of the test subjects, when the electrodes are placed on the wrist, there is virtually no change in conductance. The curve from the measurement on the wrist is gradually rising all the way through the test with no abrupt increases in the graph when the base jump occurs. From these measurements, it is concluded that it is not possible to see when the conductance started to increase and what triggered this response.

Looking at the graph for the electrodes placed in the palm of the hand, there is a significant increase in conductance, that correlates with the stimuli. From this placement of electrodes, it is possible to read from the graph when the base jump occurred.

Test subject 1 has the wrist graph that is closest to the palm measurements in that it has a clear increase in the conductance at one point. The problem with this result is that the time when the wrist graph increases does not match with the time when the base jump starts. Therefore there is no correlation between the increase in the graph and the base jump. The increase in the graph for the wrist test was 43,43%, while the increase for the palm test was 34,38%. This is the opposite of what we expected and is also opposite to what we see for the other test subjects. This error in the measurement data can be explained by the electrodes not being firmly connected to the palm for test subject 1. For all the other test subjects the graph is relatively flat. The graph for test subjects 3 and 5 starts relatively low before it stabilizes at a higher and more stable level. Because the start value was quite low, the percentage change will be quite high, which is not representative of the real value.

## Confounding factors

Several confounding factors could have been affecting the measurement results. In this part of the discussion some of these are mentioned and discussed:

- The emotional status of the test subjects. E.g., anxiety, discomfort, or insecurity. These are all things that could affect the results.
- The test subjects had to press the start button for the video themselves. This could have distracted the person and disturbed the results. It would have been better to just have one continuous video.
- It is not easy to know exactly what emotions were measured during the base jump. It could be fright, but it also could be excitement. Ideally, it should be different stimuli for each person, so everyone got a video of something that frightens them.
- Body movements. The base jump in the VR was designed the wrong direction, so you had to turn around to get the right view of the base jump. This resulted in a lot of body movement and could have affected the results because the electrodes were connected to the body, although we did some measurements before the testing and then we noticed that bodily movements did not have a significant impact on the results.
- Noises in the room that might affect the test object.
- Temperature changes in the room.
- Electric noise.
- Changes in the respiration rate. Sudden changes in the rate of respiration could affect EDA.
- Repetition of stimuli. Some of the test subjects did the test several times and may have built up habituation to the stimuli. When you know what to expect, it is not that scary anymore.

## Conclusion

This article was completed as a contribution to further development in medical technology within exposure therapy. The main goal was to construct instrumentation with a glove that could provide a clear graphic representation of the conductance level in the skin using two electrodes attached to the palm. By executing a comparison of electrode placement, it has been shown that measuring in the palm of the hand, will present a change in conductance far more exact than having electrodes on the wrist.
